# C-reactive protein haplotype is associated with high PSA as a marker of metastatic prostate cancer but not with overall cancer risk

**DOI:** 10.1038/sj.bjc.6605081

**Published:** 2009-05-12

**Authors:** C M Eklund, T L J Tammela, J Schleutker, M Hurme

**Affiliations:** 1 Department of Microbiology and Immunology, University of Tampere, Biokatu 6, Tampere 33520, Finland; 2 Department of Urology, Tampere University Hospital and University of Tampere, Teiskontie 35, Tampere 33520, Finland; 3 Laboratory of Cancer Genetics, Institute of Medical Technology, Biokatu 8, Tampere 33520, Finland; 4 Tampere University Hospital, Teiskontie 35, Tampere 33520, Finland

**Keywords:** prostate cancer, inflammation, immunology, CRP gene, association study

## Abstract

Growing evidence points to a role for inflammation in prostate carcinogenesis. The significance of C-reactive protein (CRP), an inflammatory and innate immunity molecule, has not been evaluated thoroughly in prostate cancer (PC). In this study of 739 Finnish patients with PC and 760 healthy men, we evaluated the associations of *CRP* genotypes and haplotypes with total PC risk and PC progression, using prostate-specific antigen (PSA) as a marker of metastatic disease. Although the haplotype frequencies were similar in patients and controls, an association between haplotype ACCCA and patients' PSA levels was found. The carriers more often had a high PSA than non-carriers (*P*=0.0002) and the SNP rs2794521 A-allele and rs1800947 C-allele carriers had a higher PSA than non-carriers (*P*=0.009 and *P*=0.0004, respectively). A trend for a younger age at diagnosis was found among the carriers of ACCCA (*P*=0.07) and the rs1800947 C-allele (*P*=0.06), as well as a trend for the latter to have more likely metastases (*P*=0.06), but not after Bonferroni correction (*α*=0.00208). This is the first study to suggest association between PSA and *CRP* variants in PC and, therefore, further studies are warranted. *CRP* alleles previously found to protect against increased CRP levels are now suggested to be associated with metastatic PC, indicated by elevated PSA.

Prostate cancer (PC) is the most common non-cutaneous cancer in men in western countries. Prostate cancer is a very heterogeneous and complex disease, and its pathogenesis involves both hereditary and environmental components. The histological and clinical heterogeneity of PC is also reflected at the molecular level; it is still unknown if PC is caused by a single series of genetic events or whether PC is really composed of genetically distinct subgroups. Growing evidence points towards the role of chronic inflammatory states in PC (de Visser *et al*, 2006; De Marzo *et al*, 2007). Chronic inflammation has long been linked to cancers such as stomach, liver and large intestine cancers, for which the causative role of inflammation has already been established (Ames *et al*, 1995; Lu *et al*, 2006). In PC, inflammation is probably triggered by infectious agents or exposure to environmental factors, such as dietary-derived toxins or a combination of both. Other potential initiating events of inflammation include urine reflux, physical and chemical trauma and oestrogens. The recently proposed hypothesis for PC carcinogenesis suggests that the prostate is first damaged by these triggering factors that then lead to the development of chronic inflammation and regenerative ‘risk lesions’, that is proliferative inflammatory atrophy (PIA) (De Marzo *et al*, 1999). Furthermore, damage to the epithelia may cause a loss of tolerance to normal prostatic antigens, resulting in a self-perpetuating autoimmune reaction. Histologically, most prostatic lesions containing either acute or chronic inflammatory infiltrates are associated with atrophic epithelium or focal epithelial atrophy (De Marzo *et al*, 1999). Morphologically, transitions from atrophic epithelium to adenocarcinoma and transitions from PIA to high-grade prostatic intraepithelial neoplasia have been observed (De Marzo *et al*, 1999; Putzi and De Marzo, 2000; Montironi *et al*, 2002).

C-reactive protein (CRP) is a widely used marker for the detection of inflammation and tissue damage. It is part of the soluble innate immunity system and belongs to the family of pattern-recognition molecules, which are able to bind foreign molecules for phagocytosis. After binding to a ligand, CRP binds to Fc*γ* receptors on phagocytic cells and thus mediates ligand elimination from the body. C-reactive protein is also able to activate the complement cascade by binding C1q. The known ligands for CRP are chromatin, histones, fibronectin, laminin and small nuclear ribonuclear particles (Stein *et al*, 2000). The ability of CRP to bind nuclear antigens, as well as its ability to increase the clearance of host apoptotic and necrotic cells, has led to the theory that CRP may prevent autoimmunity (Szalai, 2004). According to a recent systemic review of the association between serum CRP and cancer, CRP concentrations are usually higher in patients with cancer than in healthy controls, but the association seems to be site specific (Heikkila *et al*, 2007). The existing literature also suggests that CRP could hold a function as a prognostic marker for metastasised or androgen-independent PC (Beer *et al*, 2008; Nakashima *et al*, 2008), although the overall serum CRP concentrations of patients with PC seem not to be increased compared to those of healthy controls (Platz *et al*, 2004; Siemes *et al*, 2006; Trichopoulos *et al*, 2006; Heikkila *et al*, 2007, 2008).

Inflammation is a very complex process involving hundreds of genes. Therefore, many genes in the inflammatory pathways may contribute to the development of PC and are logical candidates for genetic determinants of PC risk (Sun *et al*, 2007). Indeed, several genes of innate immunity have already been associated with PC, such as the ribonuclease L (*RNASEL*) (Carpten *et al*, 2002), macrophage scavenger receptor 1 (*MSR1*) (Xu *et al*, 2002), members of the toll-like receptor (*TLR*) family (Zheng *et al*, 2004), macrophage inhibitory cytokine-1 *(MIC-1)* (Lindmark *et al*, 2004) and interleukin-1 receptor antagonist *(IL1RN)* (Lindmark *et al*, 2005). The *CRP* gene, which is located at chromosome site 1q23, an area previously linked to PC (Smith *et al*, 1996), consists of two exons and an intervening intron and is 2.5 kbp in length. Despite the suggested function for CRP as a prognostic marker, the significance of inherited *CRP* variation in PC has not been well studied. This prompted us to evaluate the possible function of *CRP* in advanced PC, as well as to find potential functional *CRP* SNPs relevant to PC. Here, we report an association study of *CRP* genotypes and haplotypes (haplotype composed of SNPs rs2794521, rs3091244, rs1800947, rs1130864 and rs1205) to evaluate the relationship between PC progression and the innate immunity gene *CRP* using samples from the Finnish population.

## Materials and methods

### Patients with PC and controls

DNA samples were taken from 739 consecutive patients with PC diagnosed between the years 2000 and 2003 and treated in the Pirkanmaa Hospital District Area, Finland. The detailed collection of the patients have been described elsewhere (Schleutker *et al*, 2000). In brief, the patients were primarily referred to the Department of Urology in Tampere University Hospital based on their symptoms. The clinical characteristics collected include age at diagnosis, PSA at diagnosis, tumour-metastasis stage and Gleason score. For pathological analyses biopsy samples were used primarily, but if the patient underwent radical prostatectomy and the specimen was histologically different form the original biopsy sample it was used instead. For the association analysis, the patients were divided into two subgroups based on their tumour Gleason score: <7 and >7. The lower group included tumours with a Gleason score of 7, pattern 3+4 and the upper group were those with a Gleason score of 7, pattern 4+3. Serum PSA levels were measured by the DELFIA (PerkinElmer, Wallac, Turku, Finland) method. Because only 306 men underwent a bone scan to evaluate the presence of metastases at diagnosis and of these 61 were diagnosed with metastases, PSA was used as a surrogate marker for PC stage. Thus, PSA concentration was analysed both as a continuous variable and as a categorical variable in three PSA categories: below 20 ng ml^−1^, between 20 and 100 ng ml^−1^ and greater than 100 ng ml^−1^. These categories were selected because patients with PC with a PSA over 20 ng ml^−1^ are considered high-risk patients compared to patients with a PSA below 20 ng ml^−1^, and patients with a PSA over 100 ng ml^−1^ harbour a very high risk of bone metastatic disease. The patients were classified as not having metastases only if a bone scan had been performed and no metastases found (*n*=245).

The controls were 760 anonymous healthy male blood donors with an age range of 18–65 years and were from the Helsinki, Tampere or Turku areas. The blood samples were collected by the blood centre of the Finnish Red Cross, Finland. Both the patients and controls were of same ethnic origin (Finnish Caucasians), and were gathered from the same region with a relatively homogenous population structure (the western Finns, i.e. the early settlement region) and without any statistically proven genetic drift event. Previously, no significant differences in allele frequency have been detected between our control sets, so they have been considered homogenous and combined into one set (Baffoe-Bonnie *et al*, 2005; Seppala *et al*, 2007). Written informed consent was obtained from all the patients and the research protocols were approved by the ethical committee of the Tampere University Hospital.

### SNP selection, genotyping and haplotyping

DNA samples, from 10 ml of peripheral blood, were prepared using the Wizard Genomic DNA Purification Kit (Promega Corporation, Madison, WI, USA), according to the instructions of the manufacturer. TagSNPs, a set of informative common CRP SNPs, were chosen based on earlier publications of CRP SNPs or on CRP SNPs deposited in the HapMap database using SNPtagger (de Bakker *et al*, 2005). The tagSNPs were referenced in dbSNP with the following accession numbers: −717 (rs2794521), −286 (rs3091244), +1059 (rs1800947), +1444 (rs1130864) and +1846 (rs1205). The DNA samples were genotyped using the fluorogenic 5′ nuclease assay in combination with specific TaqMan MGB probes using the ABI Prism 7900HT Sequence Detection System (Applied Biosystems, Foster City, CA, USA). For SNP +1059, a commercially available kit was used (Assay-On-Demand, C_177490; Applied Biosystems) with standard conditions. For SNPs −717, +1444 and +1846, the nucleotide sequences of the primers and allele-specific probes, labelled with reporter dyes, were designed from sequences deposited in the GenBank database using the Assay-by-Design tool and were synthesised by Applied Biosystems. SNP −286 was genotyped as previously described (Carlson *et al*, 2005), except that the A-allele detecting probe had a final concentration of 0.6 *μ*M and no betaine was used. The PCRs with no-template controls were performed in 384-well plates in a total volume of 5 *μ*l in accordance with the manufacturer's standard protocol. The end-point reading of the fluorescence was measured using the allelic discrimination analysis module, resulting in a clear identification of three (six for SNP −286) genotypes.

The haplotype estimation was carried out using the PHASE v2.0.2 programme, which is based on a Bayesian statistical method for reconstructing haplotypes from population genotype data, and lists the most probable haplotypes for each individual (Stephens and Scheet, 2005).

### Statistical analysis

Statistical analysis was performed using STATISTICA 6.1 (StatSoft Inc., Tulsa, OK, USA). Comparison of haplotype frequencies between patients and controls was carried out by Fisher's exact test. Genotype and haplotype association with demographic, clinical and pathological features of the disease was tested with Mann–Whitney *U*-test, Pearson's *χ*^2^-test and Fisher's exact test. The Bonferroni correction was applied to adjust the significance level in multiple comparisons. The genotype and haplotype associations with features of PC were regarded as statistically significant if the obtained two-tailed *P*-value was less than *α*=0.00208 (*α*-level 0.05 divided by the number of tested haplotypes (*n*=4) multiplied by the number of end points (*n*=6); 0.05/(4 × 6)=0.00208).

## Results

### Patient characteristics, genotype and haplotype frequencies

Characteristics of the 739 patients with PC are described in Table 1. The genotype frequencies of all SNPs were in Hardy–Weinberg equilibrium, except for SNP −286 in controls (*P*=0.03). There were no differences in the genotype frequencies of any SNP between the patients and controls (−717, *P*=0.07; −286, *P*=0.60; +1059, *P*=0.48; +1444, *P*=0.25 and +1846, *P*=0.32) and the frequencies were similar to those previously published in young healthy Finnish individuals (data not shown) (Eklund *et al*, 2008).

Table 2 shows the haplotype frequencies for patients and controls. Of the 739 patients and 760 controls included in the haplotype estimation procedure, the programme yielded reliable results for 728 (98.5%) patients and 744 (97.9%) controls, which were subsequently used in haplotype analyses. The 5 SNPs formed 12 haplotypes in patients with PC and 9 in controls. No differences in the haplotype frequencies between the cases and controls were observed (Table 2), except for haplotypes ATGCA (*n*=8, *P*=0.037) and GCCCG (*n*=9, *P*=0.002). However, due to the very low frequency of these particular haplotypes, the results of the hypothesis testing were prone to being affected by even small variations in the data, leaving open the possibility for positive findings by chance.

### Haplotype and individual SNP associations

We analysed whether the carrier status of the four common haplotypes (frequency >5%) was associated with the clinicopathological features of the patients with PC: PSA levels (Table 3), T stage, M stage, Gleason score and age at diagnosis (online Supplementary Table 4).

Although PSA acts as a continuous variable in PC, we wanted to scrutinise the distribution of CRP haplotype frequencies among different PSA concentrations and, thus, compared three categories. The categories for PSA were selected based upon previous findings that patients with PSA greater than 20 ng ml^−1^ have a high risk and patients with PSA greater than100 ng ml^−1^ have a very high risk of bone metastatic disease compared to those with PSA less than 20 ng ml^−1^ (Oesterling, 1991; Rana *et al*, 1992). Prostate-specific antigen greater than 100 ng ml^−1^ has been found to be the single most important indicator of metastatic disease, with a predictive value of 100% (Rana *et al*, 1992), whereas the negative predictive value of PSA less than 20 ng ml^−1^ has been found to be about 99% (Oesterling, 1991). In our patient group, less than half (41.6%; *n*=306) of the patients underwent a bone scan; of these, only 8.3% (*n*=61) were found to have metastases. For the patients with rare haplotypes, statistical tests on association between metastasis and rare haplotypes were significantly underpowered, and we had to employ an alternative method of analysis, that is categorical PSA analysis with the assumption that patients with PSA greater than 100 ng ml^−1^ have a greater risk of bone metastatic disease.

We found that carriers of haplotype ACCCA were significantly more likely to have PSA values over 100 ng ml^−1^ at the time of diagnosis than the ACCCA non-carriers (*P*=0.0002, Table 3). There was also a trend for these carriers to be younger at the time of diagnosis and to have metastases more often (*P*=0.07 and *P*=0.12, respectively; Supplementary Table 4), but these trends were negligible after the Bonferroni correction (*α*=0.00208). Also, carriers of the haplotype GCGCG were more likely to have PSA values over 20 ng ml^−1^ than the non-carriers (*P*=0.03, Table 3), but this result was not significant after the Bonferroni correction. The other haplotypes did not associate with any of the studied clinicopathological features.

The *CRP*+1059G>C silent base substitution in exon two was associated with PSA levels. Carriers of the minor allele C were more likely to have high PSA levels over 100 ng ml^−1^ than were GG homozygotes (*P*=0.0004, Table 3). The carriers were also more likely to have metastases and have a lower age at diagnosis (*P*=0.06 for both, online Supplementary Table 5), but the result was not significant after the Bonferroni correction. Other SNPs also had some significant associations with PSA or Gleason score, but not after the Bonferroni correction. The promoter region SNP −717 was associated with PSA levels so that the carriers of the major allele A had higher PSA levels than the non-carriers in linear analysis (9.1 *vs* 6.4 ng ml^−1^, *P*=0.009, Table 3). The other promoter region SNP, the three-allelic *CRP* −286, was associated with Gleason score; the carriers of the major allele C more often had a Gleason score under 7 than the non-carriers (*P*=0.03; Supplementary Table 7), showing a possible protective effect for this allele. The individual effects of the *CRP* SNPs on the clinicopathological features of PC are shown in online supplement tables (online Supplementary Tables 5–7). No statistically significant associations were found for SNPs +1444 and +1846 (data not shown).

## Discussion

Chronic inflammation/chronic inflammatory states, triggered by infectious agents or other environmental factors, have been suggested to underlie about 20% of all human cancers in adults (Ames *et al*, 1995; Coussens and Werb, 2002). Emerging evidence has also pointed to a possible function for inflammation in the pathogenesis of PC and recently a function for CRP as a prognostic marker in PC has been suggested (Beer *et al*, 2008; Nakashima *et al*, 2008). Here, we evaluated the function of an innate immunity inflammatory candidate gene, *CRP*, in PC risk and in the clinical characteristics of the disease by genotyping and haplotyping five tagging *CRP* SNPs, −717, −286, +1059, +1444 and +1846, and sought for possible functional *CRP* SNPs by means of association analysis.

Our results showed that the genotype and haplotype frequencies were similar in patients and controls, except for the very rare haplotypes, GCCCG (*n*=9) and ATGCA (*n*=8). Due to very low frequency, type I error cannot be totally excluded. However, even larger populations should be tested before totally rejecting the possibility that there are, in fact, undetected differences between patients and controls in the most common haplotypes, although none were found in our study.

Our results showed an association between *CRP* haplotypes/genotypes and patients' PSA levels. The fourth most common *CRP* haplotype, ACCCA, was associated with high PSA level in the categorical analysis, but not in the continuous variable analysis, which suggests that the association is non-linear in nature. In the categorical analysis, we found that the carriers of the haplotype ACCCA more often had PSA levels over 100 ng ml^−1^ when compared to the non-carriers (*P*=0.0002), and the result remained unchanged after Bonferroni correction for multiple comparisons (*α*=0.00208). However, we did not find that there was a significantly higher proportion of the ACCCA+ carriers with metastatic disease compared to non-carriers when analysing bone-scan-verified patients (*P*=0.12). However, as mentioned, the number of patients with verified metastases was very low. Moreover, two of the SNP alleles embedded in the ACCCA haplotype, −717 allele A and +1059 allele C, were also individually associated with high PSA levels, but that result did not remain significant after the conservative Bonferroni correction. Furthermore, in our study, the tendency of +1059 C-allele carriers to have metastases more often and to be younger at the time of diagnosis when compared to GG homozygotes (*P*=0.06 for both; Supplementary Table 5) points to a function for *CRP* haplotype/genotype in actual tumour growth and disease progression, but this result was also not significant after Bonferroni correction. Taken together, we found some evidence pointing towards an association between ACCCA haplotype and metastatic disease, but further studies should scrutinise this connection before any solid conclusions can be made.

The haplotype ACCCA and some of its alleles have previously been associated with low CRP levels in Finns (Eklund *et al*, 2008) as well as in other populations, (Carlson *et al*, 2005; Miller *et al*, 2005; Crawford *et al*, 2006; Suk Danik *et al*, 2006) although not all of the SNPs of the haplotypes used in these studies were exactly the same. According to a recent meta-analysis with well-adjusted CRP values, systemic CRP concentrations seem not to be associated with PC as such (Heikkila *et al*, 2007). However, two recent studies suggested that circulating CRP could hold a function as a prognostic marker for metastasised or androgen-independent PC (Beer *et al*, 2008; Nakashima *et al*, 2008), contradicting our results. In our study, we did not put much weight on circulating CRP because of the problem that CRP is heavily confounded by demographic, metabolic and socioeconomic factors, not to mention acute infections, everyday traumas, given therapies etc. Genetic variation, on the other hand, represents a consistent inherited factor in CRP determination throughout life, free of confounding. Even though elevated CRP could be associated with some forms of PC in the above-mentioned studies, the reason for this association might not be causal but due to confounding. This is the reason we decided to relay on inherited variation in *CRP* as a measure of lifetime exposure to either low or high levels (low/high-exposing alleles determined in before hand in young, healthy adults with careful adjustment for confounding factors).

Why would then a *CRP* haplotype previously associated with low CRP now associated with increased PSA levels and a possible increased risk of having metastases?

It is possible that an impaired defence mechanism, perhaps in the form of locally reduced CRP response in the tumour, could result in excess cellular fragments and prolonged inflammation in the tumour because CRP cannot function sufficiently to remove all cellular debris and/or apoptotic cells in the prostatic lesions. If we consider early prostate neoplasia progression, where repeated bouts of injury and cell death to the prostate epithelium occur as a result of the action of inflammatory cells in response to pathogens or autoimmune disease (phagocytic cells at inflammatory sites generate reactive oxygen and nitrogen species, which damage cells and the genome) or from direct injury from circulating carcinogens/toxins in the urine, the injury caused to the epithelium triggers a restorative epithelial cell proliferation to replace the damaged cells (Coussens and Werb, 2002; De Marzo *et al*, 2007). The morphological and biological manifestations of this injury are focal atrophy or PIA, an increase in proliferation and a massive increase in epithelial cells possessing a phenotype intermediate between basal cells and mature luminal cells (De Marzo *et al*, 1999; Nelson *et al*, 2003; van Leenders *et al*, 2003). In this situation, inadequate removal of cellular debris and/or apoptotic cells by CRP and phagocytic cells could further increase inflammation or even result in a self-perpetuating autoimmune reaction. This would further increase inflammation and the presence of immune cells and cytokines, leading to a vicious circle of increasing infiltration of immune cells and inflammation, that is chronic inflammation. All of these phases show an important connection between inflammatory processes and induction of growth of pre-neoplastic and neoplastic lesions in the prostate. In the prostatic tissue, local CRP concentration in the microenvironment, instead of the systemic CRP, could be more important in the resolution of the inflammatory response because the ‘high’ CRP producers (by genotype) are more capable of resolving the reaction and thus have an advantage compared to subjects with ‘low’ CRP producers. Monocytes and T cells have been shown to produce CRP *in vitro* (Haider *et al*, 2006), and at least epithelial cells of the respiratory tract are capable of CRP expression and secretion (Gould and Weiser, 2001). If local inflammation resolution is dysregulated, the cellular response to the pattern of chronic inflammation changes, such that macrophages and other inflammatory cells generate a great amount of growth factors, cytokines and reactive oxygen species that may cause DNA damage and predispose the patient to neoplasia.

Before our analysis, only one association study between *CRP* and PC was reported. In the paper by Siemes *et al* (2006), an association was sought between PC and three *CRP* 3′ untranslated region SNPs, +1444, +1846 and +2911, but no association was found. However, in the same study, a positive association between +1846 AA homozygotes and risk of lung cancer was observed (OR 2.6; 95% CI 1.6–4.4). In another study of elderly people, an association between *CRP* haplotype B, bearing the +1846 A-allele, and cancer-related death was found, but the cancer cases were not analysed separately according to site (Hindorff *et al*, 2008). This allele A is also part of the high-PSA-associated ACCCA haplotype in our study. Limitations of our study included the use of anonymous blood bank controls, in which only sex and age range are known. The controls were relatively young and a small fraction of them will probably be affected with PC at a later age (cumulative crude probability up to 84 years of age is 8.5% according to Finnish Cancer Registry), which might attenuate the effects seen in the study. However, control blood samples were collected from the same geographic regions as the cases (all Finnish Caucasians) and no significant differences in allele frequency have been detected among our control sets. Significant differences were neither found between blood donors and healthy young Finnish non-blood donors. Therefore, they provide an unbiased survey of population genotype frequencies.

In summary, the data presented point to a possible function of reduced local CRP response in metastatic PC aetiology. The possession of the CRP haplotype ACCCA, associated previously with low CRP concentration and now with high PSA levels, could be taken into consideration as a part of patient prognosis in PC. However, further studies of the function of *CRP* in prostate carcinogenesis are warranted, using even larger study groups from other populations, before any solid conclusions about the function of *CRP* genetics in PC are made.

## Figures and Tables

**Table 1:** Demographic data of patients with prostate cancer

**Clinical/pathological category**	***n* (%)**
*Age at diagnosis*	
Mean±s.d.	68.3±8.7
	
*Stage*	
T stage	
T_1_–T_2_ (organ-confined)	547 (74.3)
T_3_–T_4_ (extracapsular)	189 (25.7)
M stage	
0 (no metastasis)	245 (33.3)
1 (metastasis)	61 (8.3)
Prostate-specific antigen	
<20 ng ml^−1^	584 (79.8)
⩾20, ⩽100 ng ml^−1^	100 (13.7)
>100 ng ml^−1^	48 (6.6)
	
*Grade*	
Gleason score^a^	
<7	487 (69.8)
>7	211 (30.2)

a <7 includes Gleason score 7 with pattern 3+4, and >7 includes Gleason score 7 with pattern 4+3.

**Table 2:** The *CRP* haplotype frequencies in patients with PC and in controls

	**Patients with PC**		**Controls**		
**Haplotype**	***n***	**Frequency**	***n***	**Frequency**	***P*-value^a^**
1. ATGTG	519	0.356	553	0.372	0.400
2. ACGCA	417	0.286	431	0.290	0.871
3. GCGCG	335	0.230	305	0.205	0.108
4. ACCCA	78	0.054	95	0.064	0.241
5. AAGCG	66	0.045	82	0.055	0.238
6. ACGCG	15	0.010	18	0.012	0.727
7. GCCCG	9	0.006	0	0	0.002
8. ATGCA	7	0.005	1	0.001	0.037
9. ACGTG	4	0.003	1	0.001	0.213
10. GTGCG	3	0.002	0	0	0.121
11. AACCG	2	0.001	0	0	0.245
12. ATGCG	1	0.001	2	0.001	1.0
*n* (total)	1456		1488		

The frequency calculations are based on the number of chromosomes.

a Fisher's exact test.

**Table 3:** Patients' PSA levels according to *CRP* haplotype/genotype carrier status

			**PSA categorical (ng ml**^−1^)			
**Genetic variable**	**PSA median (ng ml**^−1^)	***P*-value^a^**	**<20**	**⩾20 ⩽100**	**>100**	***P*-value^a^**
ATGTG+	8.9 (0.5–15720)	0.78	332 (79.4)	61 (14.6)	25 (6.0)	0.67
ATGTG−	8.8 (0.5–8992)		243 (80.4)	38 (12.6)	21 (7.0)	
ACGCA+	8.8 (0.8–8992)	0.43	284 (80.0)	50 (14.1)	21 (5.9)	0.86
ACGCA−	8.9 (0.5–15720)		291 (79.7)	49 (13.4)	25 (6.8)	
GCGCG+	8.9 (0.5–15720)	0.88	226 (77.7)	51 (17.5)	14 (4.8)	0.03
GCGCG−	8.8 (0.5–8992)		349 (81.4)	14 (4.8)	32 (7.4)	
ACCCA+	8.5 (2.3–1750)	0.65	59 (81.9)	2 (2.8)	11 (15.3)	0.0002
ACCCA−	8.9 (0.5–15720)		516 (79.6)	97 (15.0)	35 (5.4)	
+1059 GG	9.1 (0.50–15720)	0.67	513 (79.4)	97 (15.0)	36 (5.6)	0.0004
+1059 GC+CC	8.6 (2.3–2200)		68 (81.9)	3 (3.6)	12 (14.5)	
−717 AA+AG	9.1 (0.5–15720)	0.009	546 (79.4)	97 (14.1)	45 (6.5)	0.44
−717 GG	6.4 (1.5–5959)		36 (85.7)	3 (7.1)	3 (7.1)	
−286 CC+CT+CA	8.8 (0.5–8992)	0.34	434 (78.6)	82 (14.9)	36 (6.5)	0.59
−286 TT+TA	8.6 (0.5–1272)		91 (82.7)	14 (12.7)	5 (4.5)	

*CRP* haplotype is based on SNPs −717A>G, −286C>T>A, +1059G>C, +1444C>T and +1846G>A.

GCGCG+ =carrier of haplotype GCGCG.

GCGCG− =non-carrier of haplotype GCGCG.

a The Bonferroni-corrected significance level *α* is 0.05/(6 × 4)=0.00208, that is the significance level *α*=0.05 is divided by the number of end points (6) multiplied by the number of tested haplotypes (4).
