# Supplementary material for: C-reactive protein haplotype is associated with high PSA as a marker of metastatic prostate cancer but not with overall cancer risk
Source: Br J Cancer. 2009 May 12;100(12):1846–51. doi: 10.1038/sj.bjc.6605081 (PMC2714238; doi:10.1038/sj.bjc.6605081)
Supplement: Supplementary Table 4 [file 6605081x1.doc]

Supplement Table 4. Tumour characteristics of *CRP* haplotype carriers (+) and non-carriers (-). The *CRP* haplotype is based on SNPs -717A>G, -286C>T>A, +1059G>C, +1444C>T and +1846G>A.

| Classification | Unit/  grade | ATGTG+ ATGTG- p* ACGCA+ ACGCA- p* GCGCG+ GCGCG- p* ACCCA+ ACCCA- p* | | | | | | | | | | | |
| --- | --- | --- | --- | --- | --- | --- | --- | --- | --- | --- | --- | --- | --- |
| T class, n (%)  Metastasis,  n (%)  Gleason score, n (%)  Age at diagnosis,  mean ± SD | 1-2  3-4  No  Yes  <7  ≥7  Years | 309 (73.4)  112 (26.6)  143 (80.8)  34 (19.2)  277 (69.8)  120 (30.2)  68.3 ± 8.1 | 231 (76.2)  72 (23.8)  98 (80.3)  24 (19.7)  202 (70.4)  85 (29.6)  68.3 ± 9.4 | 0.39  0.92  0.86  0.97 | 270 (75.8)  86 (24.2)  121 (83.4)  24 (16.6)  240 (70.8)  99 (29.2)  68.3 ± 8.7 | 270 (73.4)  98 (26.6)  120 (77.9)  34 (22.1)  241 (69.3)  107 (30.7)  68.3 ± 8.7 | 0.44  0.23  0.66  0.90 | 216 (73.7)  77 (26.3)  101 (82.8)  21 (17.2)  194 (69.5)  85 (30.5)  68.4 ± 9.1 | 324 (75.2)  107 (24.8)  140 (79.1)  37 (20.9)  287 (70.3)  121 (29.7)  68.2 ± 8.3 | 0.66  0.43  0.82  0.77 | 56 (76.7)  17 (23.3)  21 (70.0)  9 (30.0)  51 (76.1)  16 (23.9)  66.5 ± 8.4 | 484 (74.3)  167 (25.7)  220 (81.8)  49 (18.2)  430 (69.4)  190 (30.6)  68.5 ± 8.7 | 0.66  0.12  0.25  0.07 |

*The Bonferroni-corrected significance level α is 0.05/(4x6) =0.00208.
