# Supplementary material for: C-reactive protein haplotype is associated with high PSA as a marker of metastatic prostate cancer but not with overall cancer risk
Source: Br J Cancer. 2009 May 12;100(12):1846–51. doi: 10.1038/sj.bjc.6605081 (PMC2714238; doi:10.1038/sj.bjc.6605081)
Supplement: Supplementary Table 5 [file 6605081x2.doc]

Supplement Table 5. Tumour characteristics according to *CRP* +1059G>C genotypes and +1059 C-allele carrier status.

| Classification | Unit/  grade | +1059GG +1059GC +1059CC | | | p* | +1059GG +1059GC+CC | | p* |
| --- | --- | --- | --- | --- | --- | --- | --- | --- |
| T class, n (%)  Metastasis, n (%)  Gleason score n, (%)  Age at diagnosis,  mean ± SD | 1-2  3-4  No  Yes  < 7  ≥ 7  Years | 480 (74.1)  168 (25.9)  221 (81.5)  50 (18.5)  429 (69.4)  189 (30.6)  68.5 ± 8.6 | 61 (75.3)  20 (24.7)  22 (66.7)  11 (33.3)  54 (73.0)  20 (27.0)  66.5 ± 9.1 | 4 (100)  0  1 (100)  0  4 (100)  0  69.3 ± 4.6 | 0.49  0.12  0.35  0.14 | 480 (74.1)  168 (25.9)  221 (81.5)  50 (18.5)  429 (69.4)  189 (30.6)  68.5± 8.6 | 65 (76.5)  20 (23.5)  23 (67.6)  11 (32.4)  58 (74.4)  20 (25.6)  66.6 ± 8.9 | 0.63  0.06  0.36  0.06 |

* The Bonferroni-corrected significance level α is 0.05/(4x6)=0.00208.
