# Supplementary material for: C-reactive protein haplotype is associated with high PSA as a marker of metastatic prostate cancer but not with overall cancer risk
Source: Br J Cancer. 2009 May 12;100(12):1846–51. doi: 10.1038/sj.bjc.6605081 (PMC2714238; doi:10.1038/sj.bjc.6605081)
Supplement: Supplementary Table 6 [file 6605081x3.doc]

Supplement Table 6. Tumour characteristics according to *CRP*-717A>G genotype and allele A carrier status.

| Classification | Unit/  Grade | -717AA -717AG -717GG | | | p* | -717AA+AG -717GG | | p* |
| --- | --- | --- | --- | --- | --- | --- | --- | --- |
| T class, n (%)  Metastasis, n (%)  Gleason score, n (%)  Age at diagnosis, mean ± SD | 1-2  3-4  No  Yes  <7  ≥7  Years | 321 (74.7)  109 (25.3)  139 (78.1)  39 (21.9)  285 (69.9)  123 (30.1)  68.2 ± 8.3 | 190 (72.8)  71 (27.2)  95 (84.1)  18 (15.9)  168 (68.3)  78 (31.7)  68.4 ± 9.3 | 34 (79.1)  9 (20.9)  11 (73.0)  4 (27.0)  33 (76.7)  10 (23.3)  68.8 ± 8.6 | 0.65  0.37  0.54  0.88 | 511 (74.0)  180 (26.0)  234 (80.4)  57 (19.6)  453 (69.3)  201 (30.7)  68.3 ± 8.7 | 34 (79.1)  9 (20.9)  11 (73.3)  4 (26.7)  33 (76.7)  10 (23.3)  68.7 ± 8.6 | 0.46  0.50  0.30  0.71 |

* The Bonferroni-corrected significance level α is 0.05/(4x6) = 0.00208.
