# Supplementary material for: C-reactive protein haplotype is associated with high PSA as a marker of metastatic prostate cancer but not with overall cancer risk
Source: Br J Cancer. 2009 May 12;100(12):1846–51. doi: 10.1038/sj.bjc.6605081 (PMC2714238; doi:10.1038/sj.bjc.6605081)
Supplement: Supplementary Table 7 [file 6605081x4.doc]

Supplement Table 7. Tumour characteristics according to *CRP*-286C>T>A allele carrier status.

| Classification | Unit/  grade | CRP -286    All C Non-C p* All T Non-T p* All A Non-A p* | | | | | | | | |
| --- | --- | --- | --- | --- | --- | --- | --- | --- | --- | --- |
| T class, n (%)  Metastasis, n (%)  Gleason score, n (%)  Age at diagnosis, mean ± SD | 1-2  3-4  No  Yes  <7  ≥7  Years | 415 (74.9)  139 (25.1)  184 (80.7)  44 (19.3)  374 (71.1)  152 (28.9)  68.2 ± 8.8 | 77 (70.0)  33 (30.0)  86 (78.2)  24 (21.8)  63 (60.6)  41 (39.4)  68.2 ± 7.9 | 0.28  0.70  0.03  0.95 | 286 (73.3)  104 (26.7)  134 (81.7)  30 (18.3)  254 (68.6)  116 (31.4)  68.3 ± 8.0 | 206 (75.2)  68 (24.8)  86 (78.2)  24 (21.8)  183 (70.4)  77 (29.6)  68.1 ± 9.4 | 0.59  0.47  0.64  0.84 | 43 (66.2)  22 (33.8)  21 (77.8)  6 (22.2)  42 (67.7)  20 (32.3)  68.2 ± 9.1 | 449 (75.0)  150 (25.0)  199 (80.6)  48 (19.4)  395 (69.5)  173 (30.5)  68.2 ± 8.6 | 0.12  0.73  0.77  0.95 |

*The Bonferroni-corrected significance level α is 0.05/(4x6) = 0.00208.
